# *N*-*p*-Tolyl­pyrrolidine-1-carboxamide

**DOI:** 10.1107/S1600536812013578

**Published:** 2012-04-04

**Authors:** Yu-Feng Li

**Affiliations:** a Microscale Science Institute, Department of Chemistry and Chemical Engineering, Weifang University, Weifang 261061, People’s Republic of China

## Abstract

In the title mol­ecule, C_12_H_16_N_2_O, the pyrrolidine ring has a half-chair conformation. In the crystal, mol­ecules are linked into *C*(4) chains along [001] by N—H⋯O hydrogen bonds.

## Related literature

For the medicinal properties of pyrrolidine compounds, see: Yang *et al.* (1997 ▶). For related structures, see: Köhn *et al.* (2004 ▶); Li (2011 ▶).
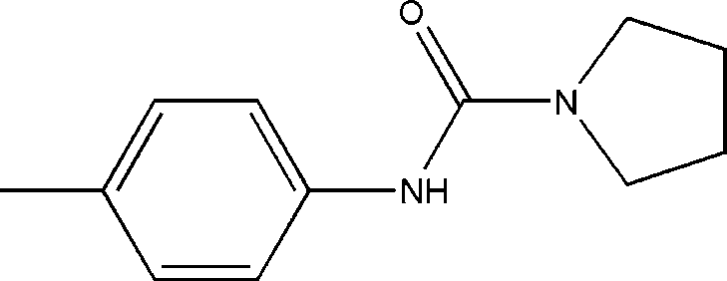


## Experimental

### 

#### Crystal data

C_12_H_16_N_2_O
*M* *_r_* = 204.27
Monoclinic, 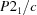

*a* = 10.264 (2) Å
*b* = 10.803 (2) Å
*c* = 10.168 (2) Å
β = 98.61 (3)°
*V* = 1114.7 (4) Å^3^
*Z* = 4
Mo *K*α radiation
μ = 0.08 mm^−1^
*T* = 293 K
0.25 × 0.22 × 0.19 mm

#### Data collection

Bruker SMART CCD diffractometer
10539 measured reflections
2553 independent reflections
1897 reflections with *I* > 2σ(*I*)
*R*_int_ = 0.028

#### Refinement

*R*[*F* ^2^ > 2σ(*F* ^2^)] = 0.052
*wR*(*F* ^2^) = 0.183
*S* = 1.14
2553 reflections
136 parameters
H-atom parameters constrained
Δρ_max_ = 0.28 e Å^−3^
Δρ_min_ = −0.23 e Å^−3^

### 

Data collection: *SMART* (Bruker, 1997 ▶); cell refinement: *SAINT* (Bruker, 1997 ▶); data reduction: *SAINT*; program(s) used to solve structure: *SHELXS97* (Sheldrick, 2008 ▶); program(s) used to refine structure: *SHELXL97* (Sheldrick, 2008 ▶); molecular graphics: *SHELXTL* (Sheldrick, 2008 ▶); software used to prepare material for publication: *SHELXTL*.

## Supplementary Material

Crystal structure: contains datablock(s) global, I. DOI: 10.1107/S1600536812013578/lh5444sup1.cif

Structure factors: contains datablock(s) I. DOI: 10.1107/S1600536812013578/lh5444Isup2.hkl

Supplementary material file. DOI: 10.1107/S1600536812013578/lh5444Isup3.cml

Additional supplementary materials:  crystallographic information; 3D view; checkCIF report

## Figures and Tables

**Table 1:** Hydrogen-bond geometry (Å, °)

*D*—H⋯*A*	*D*—H	H⋯*A*	*D*⋯*A*	*D*—H⋯*A*
N2—H2*A*⋯O1^i^	0.86	2.11	2.9301 (17)	160

Symmetry code: (i) 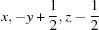.

**Fig. 1.**
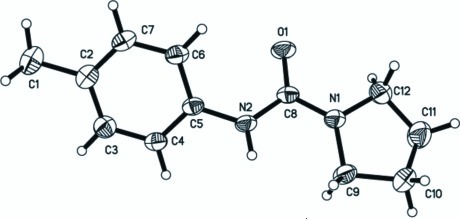
The molecular structure of the title compound showing 30% probability displacement ellipsoids.

## supplementary crystallographic information

### Comment

Pyrrolidine compounds have been shown to have medicinal properties (Yang *et al.*, 1997). The crystal structure of the title compound is presented herein. The molecular structure of the title compound is shown in Fig. 1. The pyrrolidine ring has a half-chair conformation with atoms C10 and C11 forming the twist. In the crystal, the molecules are linked into chains along [001] by intermoecular N—H···O hydrogen bonds. The structures of related compounds have already been determined (Köhn *et al.*, 2004; Li, 2011).

### Experimental

A mixture of pyrrolidine (0.1 mol), and *p*-tolylcarbamic chloride (0.1 mol) was stirred in refluxing ethanol (20 ml) for 4 h to afford the title compound (0.068 mol, yield 68%). Colourless blocks of the title compound were obtained by recrystallization of a solution of the title compound in ethanol at room temperature.

### Refinement

H atoms were fixed geometrically and allowed to ride on their attached atoms, with C—H distances = 0.93–0.97 Å; N—H = 0.86 Å and with *U*_iso_(H) = 1.2*U*_eq_(C,*N*) or 1.5*U*_eq_(C_methyl_).

### Crystal data

**Table:**

C_12_H_16_N_2_O	*F*(000) = 440
*M**_r_* = 204.27	*D*_x_ = 1.217 Mg m^−^^3^
Monoclinic, *P*2_1_/*c*	Mo *K*α radiation, λ = 0.71073 Å
Hall symbol: -P 2ybc	Cell parameters from 1897 reflections
*a* = 10.264 (2) Å	θ = 3.2–27.5°
*b* = 10.803 (2) Å	µ = 0.08 mm^−^^1^
*c* = 10.168 (2) Å	*T* = 293 K
β = 98.61 (3)°	Block, colorless
*V* = 1114.7 (4) Å^3^	0.25 × 0.22 × 0.19 mm
*Z* = 4	

### Data collection

**Table:**

Bruker SMART CCD diffractometer	1897 reflections with *I* > 2σ(*I*)
Radiation source: fine-focus sealed tube	*R*_int_ = 0.028
Graphite monochromator	θ_max_ = 27.5°, θ_min_ = 3.2°
φ and ω scans	*h* = −13→13
10539 measured reflections	*k* = −14→13
2553 independent reflections	*l* = −11→13

### Refinement

**Table:**

Refinement on *F*^2^	Primary atom site location: structure-invariant direct methods
Least-squares matrix: full	Secondary atom site location: difference Fourier map
*R*[*F*^2^ > 2σ(*F*^2^)] = 0.052	Hydrogen site location: inferred from neighbouring sites
*wR*(*F*^2^) = 0.183	H-atom parameters constrained
*S* = 1.14	*w* = 1/[σ^2^(*F*_o_^2^) + (0.0942*P*)^2^ + 0.2108*P*] where *P* = (*F*_o_^2^ + 2*F*_c_^2^)/3
2553 reflections	(Δ/σ)_max_ < 0.001
136 parameters	Δρ_max_ = 0.28 e Å^−^^3^
0 restraints	Δρ_min_ = −0.23 e Å^−^^3^

### Special details

**Table:**

Geometry. All e.s.d.'s (except the e.s.d. in the dihedral angle between two l.s. planes) are estimated using the full covariance matrix. The cell e.s.d.'s are taken into account individually in the estimation of e.s.d.'s in distances, angles and torsion angles; correlations between e.s.d.'s in cell parameters are only used when they are defined by crystal symmetry. An approximate (isotropic) treatment of cell e.s.d.'s is used for estimating e.s.d.'s involving l.s. planes.
Refinement. Refinement of *F*^2^ against ALL reflections. The weighted *R*-factor *wR* and goodness of fit *S* are based on *F*^2^, conventional *R*-factors *R* are based on *F*, with *F* set to zero for negative *F*^2^. The threshold expression of *F*^2^ > σ(*F*^2^) is used only for calculating *R*-factors(gt) *etc*. and is not relevant to the choice of reflections for refinement. *R*-factors based on *F*^2^ are statistically about twice as large as those based on *F*, and *R*- factors based on ALL data will be even larger.

### Fractional atomic coordinates and isotropic or equivalent isotropic displacement parameters (Å^2^)

**Table:**

	*x*	*y*	*z*	*U*_iso_*/*U*_eq_	
N2	0.56448 (13)	0.21960 (13)	−0.00416 (12)	0.0472 (4)	
H2A	0.5404	0.2325	−0.0877	0.057*	
O1	0.53582 (13)	0.27916 (14)	0.20476 (11)	0.0635 (4)	
C8	0.50216 (15)	0.28509 (16)	0.08300 (14)	0.0448 (4)	
N1	0.40218 (14)	0.35882 (14)	0.02833 (13)	0.0503 (4)	
C5	0.66558 (15)	0.13221 (15)	0.03298 (14)	0.0429 (4)	
C4	0.66667 (17)	0.02489 (17)	−0.04178 (16)	0.0509 (4)	
H4A	0.6007	0.0116	−0.1137	0.061*	
C6	0.76598 (17)	0.15035 (17)	0.13941 (17)	0.0530 (4)	
H6A	0.7679	0.2219	0.1905	0.064*	
C7	0.86316 (17)	0.06128 (18)	0.16900 (18)	0.0562 (5)	
H7A	0.9295	0.0746	0.2405	0.067*	
C2	0.86506 (17)	−0.04686 (16)	0.09602 (19)	0.0533 (4)	
C3	0.76466 (18)	−0.06224 (17)	−0.01046 (19)	0.0562 (5)	
H3A	0.7634	−0.1333	−0.0622	0.067*	
C9	0.34923 (18)	0.36700 (19)	−0.11286 (16)	0.0563 (5)	
H9A	0.3101	0.2890	−0.1455	0.068*	
H9B	0.4176	0.3892	−0.1648	0.068*	
C1	0.9702 (2)	−0.1433 (2)	0.1322 (3)	0.0733 (6)	
H1A	1.0299	−0.1161	0.2086	0.110*	
H1B	1.0177	−0.1554	0.0588	0.110*	
H1C	0.9300	−0.2198	0.1525	0.110*	
C12	0.3266 (2)	0.4321 (2)	0.1112 (2)	0.0659 (5)	
H12A	0.3783	0.5009	0.1520	0.079*	
H12B	0.2981	0.3816	0.1805	0.079*	
C10	0.2464 (2)	0.4672 (2)	−0.1194 (2)	0.0706 (6)	
H10A	0.2814	0.5451	−0.1461	0.085*	
H10B	0.1697	0.4455	−0.1830	0.085*	
C11	0.2112 (3)	0.4770 (3)	0.0157 (2)	0.0874 (8)	
H11A	0.1342	0.4269	0.0229	0.105*	
H11B	0.1915	0.5623	0.0351	0.105*	

### Atomic displacement parameters (Å^2^)

**Table:**

	*U*^11^	*U*^22^	*U*^33^	*U*^12^	*U*^13^	*U*^23^
N2	0.0509 (8)	0.0620 (9)	0.0274 (6)	0.0059 (6)	0.0014 (5)	0.0021 (6)
O1	0.0667 (8)	0.0948 (11)	0.0277 (6)	0.0111 (7)	0.0033 (5)	−0.0012 (6)
C8	0.0460 (8)	0.0563 (9)	0.0314 (7)	−0.0047 (7)	0.0039 (6)	0.0000 (6)
N1	0.0524 (8)	0.0602 (8)	0.0374 (7)	0.0059 (6)	0.0038 (6)	−0.0029 (6)
C5	0.0433 (8)	0.0521 (9)	0.0326 (7)	−0.0033 (6)	0.0040 (6)	0.0049 (6)
C4	0.0504 (9)	0.0588 (10)	0.0412 (8)	−0.0037 (7)	−0.0007 (7)	−0.0032 (7)
C6	0.0500 (9)	0.0587 (10)	0.0465 (9)	−0.0045 (7)	−0.0045 (7)	−0.0035 (8)
C7	0.0458 (9)	0.0646 (11)	0.0536 (10)	−0.0048 (8)	−0.0073 (7)	0.0047 (8)
C2	0.0461 (9)	0.0530 (9)	0.0609 (10)	−0.0048 (7)	0.0080 (7)	0.0134 (8)
C3	0.0567 (10)	0.0528 (10)	0.0585 (10)	−0.0036 (8)	0.0069 (8)	−0.0011 (8)
C9	0.0544 (10)	0.0718 (12)	0.0407 (8)	0.0055 (8)	0.0005 (7)	0.0075 (8)
C1	0.0562 (11)	0.0650 (12)	0.0964 (16)	0.0038 (9)	0.0036 (11)	0.0148 (11)
C12	0.0646 (12)	0.0769 (13)	0.0567 (11)	0.0122 (10)	0.0108 (9)	−0.0096 (9)
C10	0.0653 (12)	0.0741 (13)	0.0680 (13)	0.0141 (10)	−0.0041 (10)	0.0060 (10)
C11	0.0789 (15)	0.1071 (19)	0.0773 (15)	0.0339 (14)	0.0155 (12)	0.0033 (14)

### Geometric parameters (Å, º)

**Table:**

N2—C8	1.366 (2)	C2—C1	1.505 (3)
N2—C5	1.412 (2)	C3—H3A	0.9300
N2—H2A	0.8600	C9—C10	1.506 (3)
O1—C8	1.2362 (18)	C9—H9A	0.9700
C8—N1	1.351 (2)	C9—H9B	0.9700
N1—C9	1.460 (2)	C1—H1A	0.9600
N1—C12	1.461 (2)	C1—H1B	0.9600
C5—C4	1.387 (2)	C1—H1C	0.9600
C5—C6	1.392 (2)	C12—C11	1.496 (3)
C4—C3	1.379 (3)	C12—H12A	0.9700
C4—H4A	0.9300	C12—H12B	0.9700
C6—C7	1.386 (2)	C10—C11	1.477 (3)
C6—H6A	0.9300	C10—H10A	0.9700
C7—C2	1.386 (3)	C10—H10B	0.9700
C7—H7A	0.9300	C11—H11A	0.9700
C2—C3	1.388 (3)	C11—H11B	0.9700
			
C8—N2—C5	124.74 (13)	C10—C9—H9A	110.9
C8—N2—H2A	117.6	N1—C9—H9B	110.9
C5—N2—H2A	117.6	C10—C9—H9B	110.9
O1—C8—N1	121.58 (15)	H9A—C9—H9B	109.0
O1—C8—N2	122.36 (15)	C2—C1—H1A	109.5
N1—C8—N2	116.05 (13)	C2—C1—H1B	109.5
C8—N1—C9	126.04 (14)	H1A—C1—H1B	109.5
C8—N1—C12	121.21 (14)	C2—C1—H1C	109.5
C9—N1—C12	112.52 (14)	H1A—C1—H1C	109.5
C4—C5—C6	118.55 (15)	H1B—C1—H1C	109.5
C4—C5—N2	118.57 (14)	N1—C12—C11	103.79 (16)
C6—C5—N2	122.87 (15)	N1—C12—H12A	111.0
C3—C4—C5	120.61 (15)	C11—C12—H12A	111.0
C3—C4—H4A	119.7	N1—C12—H12B	111.0
C5—C4—H4A	119.7	C11—C12—H12B	111.0
C7—C6—C5	119.73 (16)	H12A—C12—H12B	109.0
C7—C6—H6A	120.1	C11—C10—C9	106.14 (17)
C5—C6—H6A	120.1	C11—C10—H10A	110.5
C2—C7—C6	122.44 (16)	C9—C10—H10A	110.5
C2—C7—H7A	118.8	C11—C10—H10B	110.5
C6—C7—H7A	118.8	C9—C10—H10B	110.5
C7—C2—C3	116.75 (16)	H10A—C10—H10B	108.7
C7—C2—C1	121.30 (17)	C10—C11—C12	107.49 (19)
C3—C2—C1	121.95 (18)	C10—C11—H11A	110.2
C4—C3—C2	121.92 (17)	C12—C11—H11A	110.2
C4—C3—H3A	119.0	C10—C11—H11B	110.2
C2—C3—H3A	119.0	C12—C11—H11B	110.2
N1—C9—C10	104.03 (16)	H11A—C11—H11B	108.5
N1—C9—H9A	110.9		

### Hydrogen-bond geometry (Å, º)

**Table:**

*D*—H···*A*	*D*—H	H···*A*	*D*···*A*	*D*—H···*A*
N2—H2*A*···O1^i^	0.86	2.11	2.9301 (17)	160

Symmetry code: (i) *x*, −*y*+1/2, *z*−1/2.
